# Histamine H1 receptor antagonist attenuates catecholamine surge and organ injury after severe burns

**DOI:** 10.3389/fendo.2023.1068925

**Published:** 2023-02-09

**Authors:** Jizhuang Wang, Chenghao Lu, Xinying Liu, Gai Zhang, Jie Zhang, Min Gao, Dan Liu, Xiong Zhang, Yan Liu

**Affiliations:** Department of burn, Shanghai Ruijin Hospital, Shanghai Jiaotong University School of Medicine, Shanghai, China

**Keywords:** histamine, cetirizine, promethazine, severe burns, catecholamine, organ injury

## Abstract

Severe burns induce a catecholamine surge, causing severe damage to the organism and raising the possibility of multisystem organ failure. Few strategies are generally acceptable to reduce catecholamine surge and organ injury post-burn. We have previously shown that histamine can amplify the catecholamine surge. In addition, promethazine, a first-generation histamine H1 receptor antagonist, alleviates catecholamine surge and organ injury after severe burns in rats. However, evidence is lacking on whether promethazine benefits patients after severe burns. Currently, sedation and analgesia (such as midazolam and fentanyl) are commonly required for patients after severe burns. It remains unclear if patients after severe burns derive clinical benefit from histamine H1 receptor antagonists combined with sedation and analgesia. This study investigates the therapeutic effect of promethazine on patients after severe burns. Moreover, we test the therapeutic effect of cetirizine, a second-generation histamine H1 receptor antagonist, combined with sedation and analgesia in rats after severe burns. We find that promethazine-pethidine treatment shows a tendency for a lower level of total bilirubin than midazolam-fentanyl in patients 7-day after severe burn. Our study confirms that cetirizine combined with midazolam and fentanyl reduces catecholamine surge and liver and lung damage after severe burns in rats; the effects are better than midazolam and fentanyl treatment. In summary, for the first time, we suggest that histamine H1 receptor antagonist has the potential clinical value of reducing liver injury in patients after severe burns. In addition, we reveal that cetirizine combined with midazolam and fentanyl may be an ideal strategy for treating severe burns.

## Introduction

Severe burns induce a vigorous harmful stress response, which leads to a significant and consistent elevation of stress hormones, including catecholamine (1–3). Catecholamine surge induces peripheral tissue ischemia, delayed wound healing, hypermetabolism, and immunosuppression (1, 4, 5). Furthermore, catecholamine surge could induce liver damage (3, 6, 7) and pulmonary edema (8). These injuries increase the probability of sepsis, multisystem organ dysfunction, and death (1, 5). Thus, it is vital to manage catecholamine surge in patients after severe burns. However, few strategies are generally acceptable to manage catecholamine surge and organ injury post-burn. Recently, we have reported that the rise of histamine level plays a vital role in catecholamine surge after severe burns while blocking histamine H1 receptor by promethazine could alleviate catecholamine surge and lung and liver injury after severe burns in rats (2). This study, for the first time, suggests the clinical value of histamine H1 receptor antagonists in reducing catecholamine levels and organ damage in patients after severe burns. Histamine H1 receptor antagonists are commonly used in clinical for allergic symptoms, such as allergic rhinitis (9) and urticaria (10). In addition, histamine H1 receptor antagonists are occasionally used for post-burn itch in hypertrophic scars in postburn patients (11). However, it remains unclear if histamine H1 receptor antagonist could alleviate organ injury after severe burns in patients.

Promethazine is a first-generation histamine H1 receptor antagonist (12). We assume other histamine H1 receptor antagonists may have the same effect. Cetirizine is a second-generation histamine H1 receptor antagonist commonly used in clinical practice. Compared with promethazine, cetirizine has fewer side effects such as sedation and drowsiness (13, 14). Thus, cetirizine may be an ideal alternative to promethazine. However, additional study is needed to clarify whether cetirizine has similar effects of regulating catecholamine levels and reducing lung and liver injury as promethazine.

Sedative and analgesic treatment (such as midazolam and fentanyl) is commonly used clinically for patients after severe burns, which helps to reduce pain, improve compliance with diagnostic and therapeutic operations, and improve the comfort of mechanically ventilated (15, 16). However, sedation and analgesia protocols are aimed more at relieving the patient’s pain but not at reducing the patient’s organ damage (17). Although studies have shown that severe burns can cause multiple organ damage, including liver and lung (18, 19), there is a lack of clinically targeted treatment to reduce organ damage in patients after severe burns. Fentanyl is an opioid analgesic; midazolam is a short-acting benzodiazepine sedative. The effects and mechanisms of fentanyl and midazolam differ from those of histamine H1 receptor antagonists. Therefore, combining histamine H1 receptor antagonists with fentanyl and midazolam may reduce the catecholamine surge and organ injury in patients after severe burns in addition to the sedative and analgesic effects. Further experimental data are required to demonstrate whether the combination of histamine H1 receptor antagonists and sedative-analgesic drugs has better efficacy than sedative-analgesic treatment.

In this study, we investigate the potential liver-protection effect of promethazine in patients after severe burns. We also evaluate the potential therapeutic value of combining midazolam, fentanyl, and cetirizine on catecholamine surge and liver and lung injury in rats after severe burns.

## Methods

### Human subjects

Clinical data were obtained from the patient’s medical records from Shanghai Jiao Tong University School of Medicine Affiliated Ruijin Hospital. Ethical approval was obtained from the independent ethics committee of Shanghai Ruijin Hospital affiliated with Shanghai Jiao Tong University, School of Medicine (2018-14). All 76 patients after severe burns (> 70% total body surface area (TBSA)) from January 1, 2017, to December 31, 2019, were collected. Patients with incomplete information, younger than 14 years, patients who were not admitted to the hospital on the day of the injury, or whose hospital stay was less than seven days were excluded (n = 25). Finally, 51 patients were included and analyzed in this study. The patients were divided into two groups according to the treatments they received: the Fen+Mid group (fentanyl + midazolam, n = 30) and the Pet+Pro group (pethidine + promethazine, n=21). Informed consent was not required owing to the retrospective nature of the study and anonymized patient records. Patient information is summarized in **Table 1**.

**Table 1:** General information of patients.

Variable	Fen+Mid	Pet+Pro	p-value
Gender			0.6259
Male	21	16	
Female	9	5	
Age (year)	46.07 ± 13.60	43.76 ± 14.40	0.5634
Total body surface area (TBSA, %)	86.10 ± 6.18	85.43 ± 9.41	0.7594

### Reagents

Cetirizine (H21023058, Northeast Pharma), Midazolam (H21023058, Northeast Pharma), and Fentanyl (H21023058, Northeast Pharma) were purchased from Northeast Pharma (Shenyang, China). Adrenaline and noradrenaline (NE) enzyme-linked immunosorbent assay (ELISA) kit (BA E-5400, LDN) was purchased from LDN (Nordhorn, German). Rat IL-1β (A1010A0301b) and IL-10 (A1010A0310) ELISA kit were purchased from BioTNT (Shanghai, China).

### Analysis of lung and liver architecture

Lung and liver architecture was analyzed as previously reported (2). Briefly, the unilateral lung of the rats was collected to measure wet weight. Then, the lung tissues were placed at 60°C until completely dry to measure the dry weight. The pulmonary wet/dry ratio means the wet weight/the dry weight. The paraffin-embedded tissue sections of the lung and liver were stained with Hematoxylin and Eosin (HE) utilizing standard techniques.

### ELISA

According to the manufacturer’s instructions, levels of Adrenaline and NE, IL-1β, and IL10 were measured *via* ELISA kits. In brief, standards or samples were added to the antibody-coated plate and incubated for 90 minutes at 37°C. The bioconjugated antibody solution, avidin-HRP solution, and TMB substrate solution were added sequentially to the microplate wells. The absorbance at 450 nm was measured within 15 min of the addition of the termination solution.

### Biochemical measurements

Rat plasma alanine aminotransferase (ALT), aspartate aminotransferase (AST), γ-Glutamine transferase (γ-GT), albumin (ALB), total bilirubin (TBIL), direct bilirubin (DBIL), creatine kinase (CK), creatine kinase isoenzyme MB (CK-MB), lactate dehydrogenase (LDH), blood urea nitrogen (BUN), and creatinine (CR) were measured using Rayto Chemray 800 Automated Chemistry Analyzer (Rayto, Shenzhen, China), according to the manufacturer’s protocol.

### Animal and severe burn models

Male Sprague Dawley rats (6–10 weeks old) were purchased from the Shanghai Laboratory Animal Center and housed at the Animal Science Center of the Shanghai Jiao Tong University, School of Medicine (SJTUSM). Rats were maintained under a 12-hour light/dark cycle at 22 °C, two rats per cage. All experimental protocols were approved by the SJTUSM Institutional Animal Care and Use Committee.

The 30% TBSA III degree scald rat model was established as reported (2). Briefly, the rats were exposed to a 30% skin surface area of their backs. The exposed skin was immersed in a 92°C water bath for 20 seconds to inflict a full-thickness burn (Rats in the Sham group were treated with 37 °C water). Then, the rats were resuscitated with saline (2 ml/kg/1%TBSA). On the day of the injury and the first day after the injury, rats in the Fen+Mid group were administered midazolam (1.5 mg/kg) and fentanyl (10 μg/kg) by intraperitoneal injection; rats in the Fen+Mid+Cet group were additionally administered cetirizine (20 mg/kg) by gavage. The rats were sacrificed on the third day after injury. Lung, liver tissues, and plasma were collected.

### Statistical analysis

All results are presented as means ± SD. Data that conform to the normal distribution was analyzed by parametric tests (Shapiro–Wilk test). Student’s t-test was used to analyze differences between the two groups, and one-way analysis of variance (ANOVA) followed by the Tukey’s post-test was used to analyze differences in multi groups. A *p*<0.05 was considered statistically significant. Data were analyzed using GraphPad Prism 9.0 (Aspire Software International).

## Results

### Promethazine + pethidine is associated with less liver injury than fentanyl + midazolam in patients after severe burns

**Figure 1:**
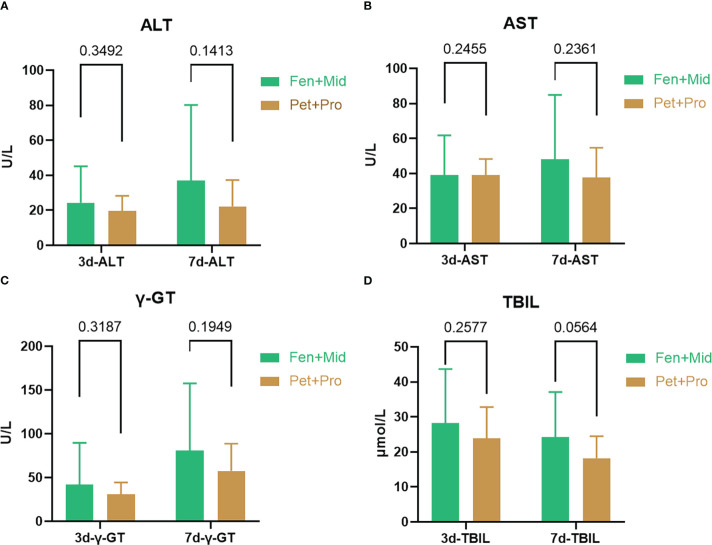
Promethazine + pethidine is associated with less liver injury than fentanyl + midazolam in patients after severe burns. The patients were divided into two groups according to the treatments they received: the Fen+Mid group (fentanyl + midazolam, n = 30) and the Pet+Pro group (pethidine + promethazine, n=21). The plasma levels of alanine aminotransferase (ALT, **(A)**), aspartate aminotransferase (AST, **(B)**), γ-Glutamine transferase (γ-GT, **(C)**), and total bilirubin (TBIL, **(D)**) on the three or seven days after patient’s admission were obtained from the patient’s medical records.

It remains unclear whether promethazine benefits patients after severe burns. We investigated the potential therapeutic effect of promethazine on patients after severe burns. At our center, there are two plans for sedation and analgesia of patients after severe burns: fentanyl + midazolam or promethazine + pethidine. The effects of different treatments on liver injury in patients after severe burns were analyzed. As the data shows, the means of plasma level of ALT (**Figure 1A**), AST (**Figure 1B**), γ-GT (**Figure 1C**), and TBIL (**Figure 1D**) on the three or seven days after the patient’s admission are both lower in the Pet+Pro group than in the Fen+Mid group. Moreover, we note a tendency for a lower level of TBIL in the Pet+Pro group than in the Fen+Mid group in patients 7-day after severe burn (*p* = 0.0564).

### Effects of fentanyl and midazolam combined with cetirizine on lung injury in rats after severe burns

**Figure 2:**
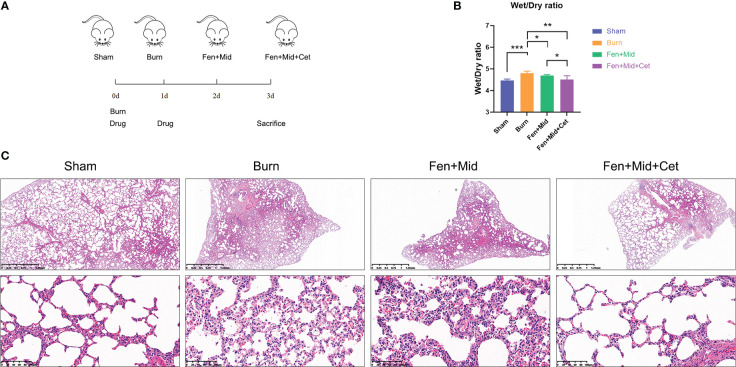
Effects of fentanyl and midazolam combined with cetirizine on lung injury in rats after severe burns. **(A)** Schematic representation of *in-vivo* study design. Ovall 24 rats were randomly divided into sham scald group (Sham, n=6), large scald group (Burn, n=6), fentanyl + midazolam group (Fen+Mid, n=6), and fentanyl + midazolam + cetirizine group (Fen+Mid+Cet, n=6). The large scald rat model was constructed on day 0. The drugs were administered on day 0 and day 1. The plasma, liver, and lung tissues of rats were collected on day 3. **(B)** The wet/dry ratio of lung tissue of rats in different groups was measured. n = 6. **(C)** The lung tissues of rats in each group were stained with HE. The scale bar indicates 1.25mm or 100 µm, respectively. *p < 0.05, ** *p* < 0.01, ***p < 0.001.

Subsequently, the corresponding *in vivo* study was designed to explore further the clinical value of midazolam and fentanyl combined with cetirizine treatment. The grouping and dosing schedule are shown in **Figure 2A**. Overall 24 rats were randomly divided into the Sham, Burn group, Fen+Mid group, and Fen+Mid+Cet group. The drug was administered on the day of scalding and on the first post-injury day, and tissue samples were collected from the rats on the third post-injury day.

The results of the rat lung tissue-related study suggested that the lung tissue wet/dry ratio was significantly higher in rats in the Burn group than rats in the Sham group (**Figure 2B**); lung tissue edema in the Burn group was evident (**Figure 2C**). The lung tissue wet/dry ratio was significantly lower in the Fen+Mid group than in the Burn group (**Figure 2B**), while there was less difference in the degree of lung tissue edema between the two groups (**Figure 2C**). In the Fen+Mid+Cet group, the lung tissue wet/dry ratio was significantly lower than that in the Fen+Mid group (**Figure 2B**), and lung tissue edema was significantly reduced (**Figure 2C**). These results suggest that fentanyl and midazolam combined with cetirizine can significantly reduce lung tissue injury in rats after severe burns, and its effect is significantly better than that of fentanyl and midazolam.

### Effects of fentanyl and midazolam combined with cetirizine on liver injury in rats after severe burns

**Figure 3:**
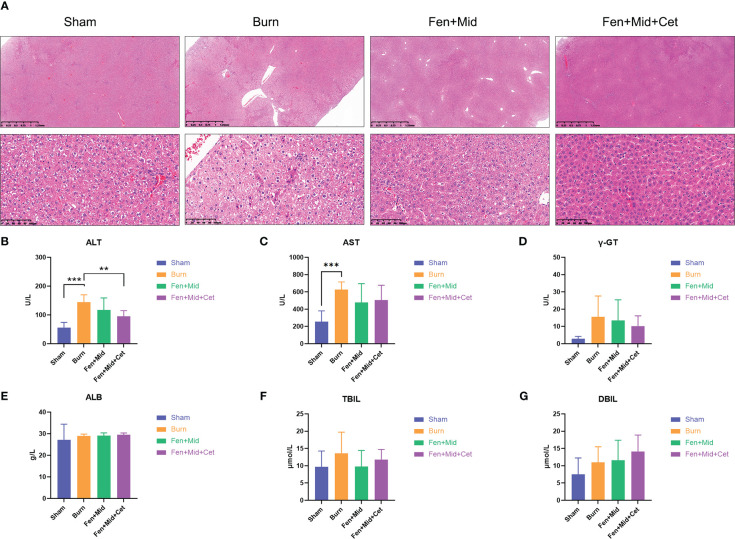
Effects of fentanyl and midazolam combined with cetirizine on liver injury in rats after severe burns. **(A)** The liver tissues of rats in large area scald group (Burn), sham scald group (Sham), fentanyl + midazolam group (Fen+Mid), or fentanyl + midazolam + cetirizine group (Fen+Mid+Cet) were stained with HE. The scale bar indicates 1.25mm or 100 µm, respectively. The plasma of rats in each group was measured with alanine aminotransferase (ALT, **(B)**), aspartate aminotransferase (AST, **(C)**), γ-Glutamine transferase (γ-GT, **(D)**), Albumin (ALB, **(E)**), Total bilirubin (TBIL, **(F)**), and Direct Bilirubin (DBIL, **(G)**) levels. n = 6, ** *p* < 0.01, ***p < 0.001.

The results of liver tissue-related studies in rats suggested that the liver tissue of rats in the Burn group was disorganized with vacuolar degeneration of hepatocytes (**Figure 3A**); plasma ALT and AST levels were significantly increased (**Figures 3B, C**). There were no significant differences in liver tissue structure and plasma ALT levels between rats in the Fen+Mid and Burn groups (**Figure 3B**). The rats in the Fen+Mid+Cet group showed significantly less disorganized liver tissue structure than those in the Burn group (**Figure 3A**); plasma ALT levels were significantly lower (**Figure 3B**). In addition, the groups had no significant differences in plasma γ-GT, ALB, TBIL, and DBIL levels (**Figures 3D–G**). These results suggested that fentanyl and midazolam combined with cetirizine could significantly reduce liver tissue injury in rats after severe burns. Its effect was superior to that of fentanyl and midazolam.

### Effects of fentanyl and midazolam combined with cetirizine on plasma catecholamine levels and inflammatory factors in rats

**Figure 4:**
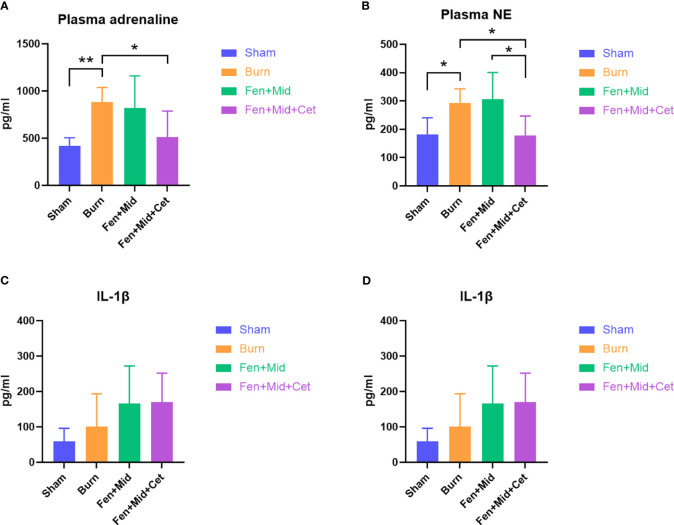
Effects of fentanyl and midazolam combined with cetirizine on plasma catecholamine levels and inflammatory factors in rats. The plasma adrenaline **(A)**, noradrenaline (NE, **(B)**), IL-1β **(C)**, and IL-10 **(D)** levels of rats in large area scald group (Burn), sham scald group (Sham), fentanyl + midazolam group (Fen+Mid), or fentanyl + midazolam + cetirizine group (Fen+Mid+Cet) were measured. n = 6, *p < 0.05, ** *p* < 0.01.

The plasma catecholamine levels of rats were significantly higher in the Burn group than in the Sham group. There was no significant difference between the plasma catecholamine levels of rats in the Fen+Mid and Burn groups. The plasma catecholamine levels of rats in the Fen+Mid+Cet group were significantly lower than those in the Burn group. (**Figures 4A, B**). These results suggest that fentanyl and midazolam combined with cetirizine can considerably reduce the catecholamine surge in rats after severe burns. Its effect is better than that of fentanyl and midazolam.

In addition, we analyzed the plasma IL-1β and IL-10 (**Figures 4C, D**) levels in each group, and the results showed no significant differences in plasma IL-1β and IL-10 (**Figures 4C, D**) levels between the groups. These results indicate that the immunomodulatory effect of cetirizine in rats after severe burns is relatively insignificant.

### Effects of fentanyl and midazolam combined with cetirizine on heart and kidney injury in rats after severe burns

**Figure 5:**
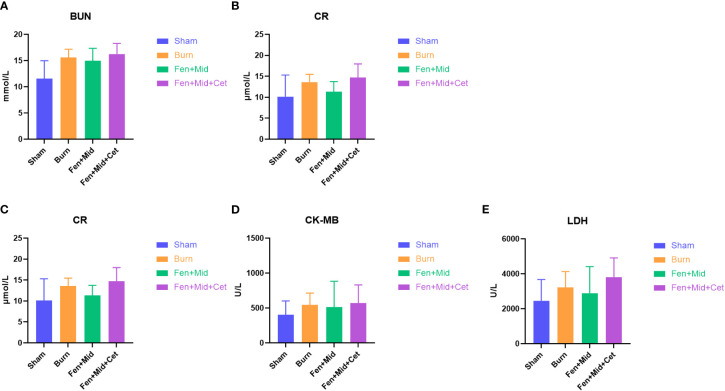
Effects of fentanyl and midazolam combined with cetirizine on heart and kidney injury in rats after severe burns. The plasma blood urea nitrogen (BUN, **(A)**), creatinine (CR, **(B)**) creatine kinase (CK, **(C)**), creatine kinase isoenzyme MB (CK-MB, **(D)**), and lactate dehydrogenase (LDH, **(E)**) levels of rats in large area scald group (Burn), sham scald group (Sham), fentanyl + midazolam group (Fen+Mid), or fentanyl + midazolam + cetirizine group (Fen+Mid+Cet) were measured. n = 6.

In addition, we analyzed the plasma BUN (**Figure 5A**), CR (**Figure 5B**), CK (**Figure 5C**), CK-MB (**Figure 5D**), and LDH (**Figure 5E**) levels in each group. The results showed no significant differences in these indicators between the groups. These results suggest that the effect of heart and kidney injury protection of cetirizine in rats after severe burns is relatively insignificant.

## Discussion

Severe burns can induce catecholamine surge and organ injury, leading to poor outcomes. Our previous study found that histamine plays a vital role in catecholamine surge after severe burns (2). We demonstrated that promethazine, a histamine H1 receptor antagonist, can reduce catecholamine surge and liver and lung injury in rats after severe burns. In this study, we explore whether promethazine can reduce liver damage in patients after severe burns for the first time. In clinical practice, sedative and analgesic treatment is generally performed for patients after severe burns (15, 17). At our center, there are two plans for sedation and analgesia of clinical patients: fentanyl + midazolam or promethazine + pethidine. Fentanyl is an opioid analgesic like pethidine (20). Midazolam is a short-acting benzodiazepine, which has the effects of anti-anxiety, sedation, hypnosis, and muscle relaxation but with side effects such as respiratory depression (21, 22). We find that promethazine-pethidine treatment showed a tendency for milder liver injury than midazolam-fentanyl in patients 7-day after severe burns. Moreover, the Pet+Pro group shows a trend for a lower level of TBIL than the Fen+Mid group in patients 7-day after severe burn (p = 0.0564). As a previous study suggested, decreasing TBIL to protect liver function may be beneficial in improving survival rate in patients (23). This result is consistent with our previous study, suggesting that histamine H1 receptor antagonists may have a protective effect on liver injury after severe burns.

The pharmacological mechanisms of fentanyl and midazolam differ from those of histamine H1 receptor antagonists. The combination of fentanyl, midazolam, and histamine H1 receptor antagonists may reduce the catecholamine surge and organ injury in patients after severe burns in addition to the sedative and analgesic effects. To further explore whether histamine H1 receptor antagonists are beneficial in reducing organ injury after severe burns, we evaluate the potential therapeutic value of combining fentanyl, midazolam, and cetirizine in the present study in rats. Cetirizine is a second-generation histamine H1 receptor antagonist with fewer side effects, such as sedation and drowsiness, than promethazine (13). Cetirizine can reduce allergic reactions and systemic capillary leakage and is clinically applied for treating urticaria, allergic rhinitis, dermatitis, and conjunctivitis (14). In addition, cetirizine is an unconventional drug for treating post-burn pruritus (24). However, the effects of cetirizine on catecholamine surge and organ protection after severe burns have not been clarified. In the present study, we investigate for the first time the protective effects of cetirizine on organ injury in rats after severe burns.

This study suggests that fentanyl and midazolam combined with cetirizine significantly reduce plasma catecholamine levels in rats after severe burns. In contrast, fentanyl combined with midazolam had no significant effect on them. This is consistent with our previous study (2), suggesting that histamine H1 receptor antagonist is efficient in reducing the catecholamine surge. For liver injury, in addition to ALT, in this study, we also measured plasma AST, γ-GT, ALB, TBIL, and DBIL levels in rats. In this study, we found no significant differences in plasma γ-GT, ALB, TBIL, and DBIL levels among the groups, while plasma ALT levels changed more significantly, which suggests that ALT is a more sensitive indicator of liver injury in rats after severe burns. In patients after severe burns, the TBIL in the promethazine + pethidine group decreased more significantly than ALT, possibly due to the different physiological characteristics of humans and rats. Combining fentanyl, midazolam, and cetirizine significantly reduced vacuolar degeneration of hepatocytes, disorganization of liver tissue, and the plasma ALT level in rats after severe burns. In contrast, fentanyl combined with midazolam had no significant effect on these aspects, which suggested that fentanyl and midazolam combined with cetirizine had a better liver protective effect than fentanyl and midazolam. In addition, we found that the wet/dry ratio of lung tissue and the degree of lung tissue edema in the rats in the fentanyl and midazolam combined with cetirizine group were significantly lower than those in the fentanyl combined with midazolam group, which indicated that fentanyl and midazolam combined with cetirizine had a better protective effect on lung tissue than fentanyl and midazolam. The above results suggest that fentanyl and midazolam combined with cetirizine can further reduce catecholamine surge and liver and lung injury after severe burns than fentanyl and midazolam, suggesting that the combination of cetirizine, fentanyl, and midazolam has important clinical application value. These results highlight the clinical value of histamine H1 receptor antagonists in reducing organ injury after severe burns.

In addition, we measured the plasma levels of pro-inflammatory factor IL-1β and anti-inflammatory factor IL-10, which reflect the level of inflammation in the body, CK, CK-MB, and LDH, which reflect the damage of cardiomyocytes, BUN, and CR, which reflect the injury of kidney, in rats. The absence of statistical differences between groups in these indicators suggests that the effects of fentanyl and midazolam combined with cetirizine in immune regulation and heart or kidney protection after severe burns are relatively insignificant or require experimental validation in larger sample sizes. Additional trials with larger sample sizes are necessary to confirm our findings.

In addition to regulating catecholamines surge, there may be other mechanisms for the effect of histamine H1 receptor antagonists in reducing organ injury. In addition to catecholamines, histamine upregulation may mediate pulmonary edema (25, 26), suggesting histamine H1 receptor antagonists may alleviate lung damage through antihistamine effects after severe burns. In addition, levocetirizine, a nonsedating H1 antihistamine, protects against lung inflammation and pulmonary edema in LPS-challenged rats (25), indicating that histamine H1 receptor antagonists may alleviate lung damage by regulating inflammation. In addition, although the correlation between histamine upregulation and liver injury after severe burns is unclear, it is also possible that histamine H1 receptor antagonists alleviate liver damage through antihistamine effects, for high histamine levels may lead to liver tissue injury (27). These facts show that histamine H1 receptor antagonists may reduce organ injury after severe burns in several aspects, highlighting their potential therapeutic value for patients after severe burns.

In conclusion, for the first time, this study suggests histamine H1 receptor antagonists may have a protective effect on liver injury after severe burns. This study demonstrates that fentanyl and midazolam combined with cetirizine significantly attenuate the catecholamine surge and liver and lung injury in rats after severe burns. These effects are superior to midazolam combined with fentanyl treatment. The experimental data suggest that the use of cetirizine in patients with severe burns has potential clinical value. It requires further clinical study data to support this clinical value. Still, it is believed that the results of this study will provide the necessary data support and ideas for further exploration and optimization of therapeutic strategies for patients after severe burns.

## Data availability statement

The original contributions presented in the study are included in the article/supplementary material. Further inquiries can be directed to the corresponding authors.

## Ethics statement

Ethical approval was obtained from the independent ethics committee of Shanghai Ruijin Hospital affiliated with Shanghai Jiao Tong University, School of Medicine (2018-14). Informed consent was not required owing to the retrospective nature of the study and anonymized patient records. The Institutional Animal Care and Use Committee of Shanghai Jiao Tong University School of Medicine reviewed and approved the animal study.

## Author contributions

JW conducted experiments and wrote the manuscript. CL assisted in experiments and statistical analysis. XL, GZ and JZ participated in animal experiments. MG and DL conducted project design and experimental guidance. XZ and YL provided technical and material support and revised the manuscript. All authors contributed to the article and approved the submitted version.
